# Extra-neural metastases in pediatric diffuse midline gliomas, H3 K27-altered: presentation of two cases and literature review

**DOI:** 10.3389/fnmol.2023.1152430

**Published:** 2023-07-20

**Authors:** Lucia De Martino, Stefania Picariello, Carmela Russo, Maria Elena Errico, Pietro Spennato, Maria Rosaria Papa, Nicola Normanno, Giuseppe Scimone, Giovanna Stefania Colafati, Antonella Cacchione, Angela Mastronuzzi, Maura Massimino, Giuseppe Cinalli, Lucia Quaglietta

**Affiliations:** ^1^Neurooncology Unit, Department of Pediatric Oncology, Santobono-Pausilipon Children's Hospital, Naples, Italy; ^2^Neuroradiology Unit, Department of Neurosciences, Santobono-Pausilipon Children's Hospital, Naples, Italy; ^3^Patology Unit, Department of Pathology, Santobono-Pausilipon Children's Hospital, Naples, Italy; ^4^Pediatric Neurosurgery Unit, Department of Pediatric Neurosciences, Santobono-Pausilipon Children's Hospital, Naples, Italy; ^5^Department of Paediatric Haematology/Oncology, Cell Therapy, A.O.R.N. Santobono-Pausilipon, Naples, Italy; ^6^Cell Biology and Biotherapy Unit, Istituto Nazionale Tumori-IRCCS “Fondazione G. Pascale”, Naples, Italy; ^7^Radiotherapy Unit, AOU San Giovanni di Dio e Ruggi d'Aragona, Salerno, Italy; ^8^Oncological Neuroradiology Unit, Department of Imaging, Istituto di Ricovero e Cura a Carattere Scientifico, Bambino Gesù Children's Hospital, Rome, Italy; ^9^Neurooncology Unit, Department of Paediatric Haematology/Oncology, Cell and Gene Therapy, Istituto di Ricovero e Cura a Carattere Scientifico, Bambino Gesù Children's Hospital, Rome, Italy; ^10^Pediatric Oncology, Fondazione IRCCS-Istituto Nazionale dei Tumori, Milan, Italy

**Keywords:** pediatric, diffuse midline glioma, H3 K27, metastases, high grade glioma, brain tumors

## Abstract

**Introduction:**

Pediatric diffuse midline gliomas (DMG), H3 K27- altered, are the most aggressive pediatric central nervous system (CNS) malignancies. Disease outcome is dismal with a median survival of less than one year. Extra-neural metastases are an unusual occurrence in DMG and have been rarely described.

**Methods and results:**

Here, we report on two pediatric patients affected by DMG with extra-neural dissemination. Their clinical, imaging, and molecular characteristics are reported here. An 11-year-old male 5 months after the diagnosis of diffuse intrinsic pontine glioma (DIPG) developed metastatic osseous lesions confirmed with computed tomography (CT) guided biopsy of the left iliac bone. The patient died one month after the evidence of metastatic progression. Another 11-year-old female was diagnosed with a cerebellar H3K27- altered DMG. After six months, she developed diffuse sclerotic osseous lesions. A CT-guided biopsy of the right iliac bone was non-diagnostic. She further developed multifocal chest and abdominal lymphadenopathy and pleural effusions. Droplet digital polymerase chain reaction (ddPCR) on pleural effusion revealed the presence of H3.3A mutation (c.83A>T, p.K28M). The patient died 24 months after the diagnosis of DMG and 3 months after the evidence of metastatic pleural effusion.

**Discussion:**

Extra-neural metastasis of DMG is a rare event and no standard therapy exists. An accurate and early diagnosis is necessary in order to develop a personalized plan of treatment. Further research is needed to gain further insights into the molecular pathology of DMG, H3K27- altered and improve the quality of life and the final outcome of patients with this deadly disease.

## 1. Introduction

Pediatric diffuse midline gliomas (DMGs), H3 K27-mutant, are a rare group of malignancies, first introduced in the 2016 World Health Organization (WHO) Classification of Tumors of the Central Nervous System (CNS) with loss of H3p.K28me3 (K27me3) and usually an H3 c.83A>T p.K28M (K27M) substitution in one of the histone H3 isoforms (CNS WHO grade 4). In the 2021 WHO Classification of Tumors of the CNS, the DMGs have been renamed to “diffuse midline glioma, H3 K27- altered” to include additional molecular changes (such as aberrant overexpression of EZHIP, or an EGFR mutation) that also result in H3 K27 alterations (Louis et al., 2016, 2021). The preferential location is the brainstem or the pons [the latter named diffuse intrinsic pontine glioma (DIPG)], or bithalamic, whereas DMGs in adolescents and adults predominantly arise unilaterally in the thalamus or in the spinal cord (Louis et al., 2021). On magnetic resonance imaging (MRI), DIPGs classically have their epicenter in the pons and typically involve >50% of its surface, often asymmetrically, with frequent encasement of the basilar artery (Steffen-Smith et al., 2014). There may be an exophytic component and/or infiltration into the midbrain, the cerebellar peduncles, and the cerebellar hemispheres. Thalamic tumors may be unilateral or bilateral, the latter being more frequent in the *EGFR*-mutant subtype (Broniscer et al., 2018). Although epidemiological data remain scant for DMG, the incidence of DIPG is estimated to be 0.54 cases per 1 million person-years overall and 2.32 cases per 1 million person-years in people aged ≤ 20 years, with no sex predilection (Mackay et al., 2017). DIPG represents 10%−15% of all pediatric brain tumors and 75% of all pediatric brainstem tumors. Thalamic DMGs are rarer, representing 1%−5% of pediatric brain tumors (25% of thalamic tumors) (Ryall et al., 2016). To date, there is no known specific genetic susceptibility for DMG, but exceptionally, DMGs may occur in the setting of a cancer predisposition syndrome such as Li–Fraumeni syndrome or mismatch repair deficiency. Independently from the location, the prognosis of DMG is poor, with a 2-year survival rate of < 10% (Mackay et al., 2017). Large autopsy-based studies of DIPG have described leptomeningeal metastasis in 40% of cases, as well as diffuse spread to involve the thalamus, the cervical cord, and even the frontal lobe (Buczkowicz et al., 2014). To date, extra-neural metastases in patients with DMG, H3 K27-altered, have been reported in 12 cases (Megan et al., 2018; Stephens et al., 2019; Bhatt et al., 2020; Handis et al., 2021; Li et al., 2021; Mohiuddin et al., 2021; Al Sharie et al., 2022; Lazow et al., 2022; Silva et al., 2022; Aftahy et al., 2023). In this study, we report two cases of pediatric DMG with extra-neural metastasis carrying H3.3K27 mutation: one patient was found to have osseous and bone marrow metastases and the second one showed multiple bone lesions, multifocal chest and abdominal lymphadenopathy, and metastatic pleural effusion.

## 2. Case reports

### 2.1. Case 1

**Figure 1:**
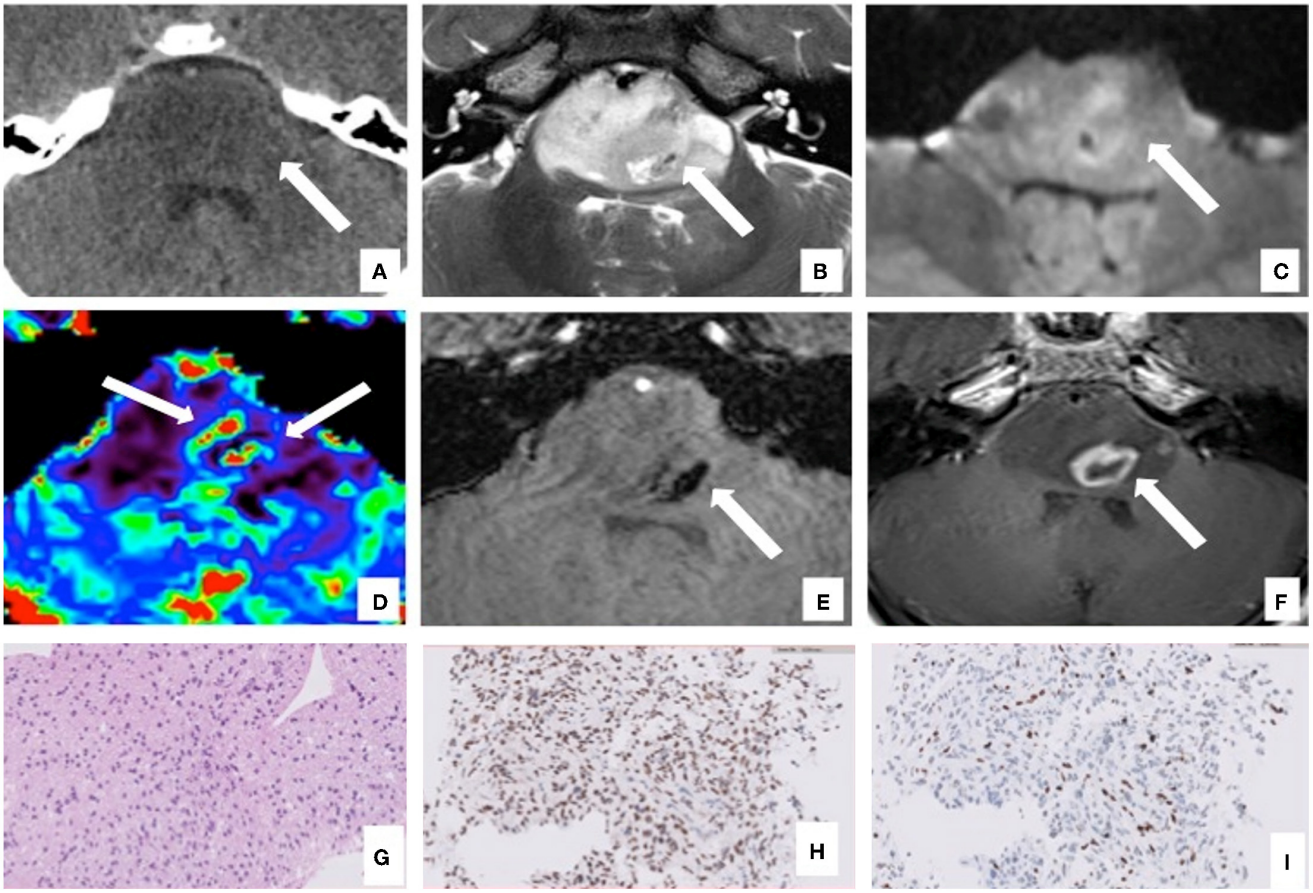
Brain computed tomography (CT), magnetic resonance imaging (MRI), and pathology of the primary site of case 1. The axial CT images show a diffuse low-density centered in the enlarged pons with the flattening of the fourth ventricle and surrounding structures [**(A)**, arrow]. Axial MRI images confirm the lesion characterized by a homogeneous high signal on T2 image **(B)** involving more than 50% of the pons with limited restricted areas on diffusion-weighted imaging [DWI, **(C)**]. The MRI also showed areas within the lesion characterized by high cerebral blood volume (CBV) values on perfusion sequences [**(D)**, arrows]. The lesion presents few necrotic components on the left side [**(B, F)**, arrow] with areas defined by a low signal on susceptibility weighted imaging (SWI) sequences due to hemosiderin deposition [**(E)**, arrow] and peripheral enhancement on T1 post-contrast sequences [**(F)**, arrow]. The T2W images also demonstrated the basilar artery encasement [**(B)**, arrowhead]. Histological examination confirmed the radiological diagnosis of DIPG, showing an infiltrative glial cell proliferation [H&E, **(G)**], which displayed the expression of H3K27M mutation **(H)** and the loss of H3K27me3 protein expression **(I)**.

**Figure 2:**
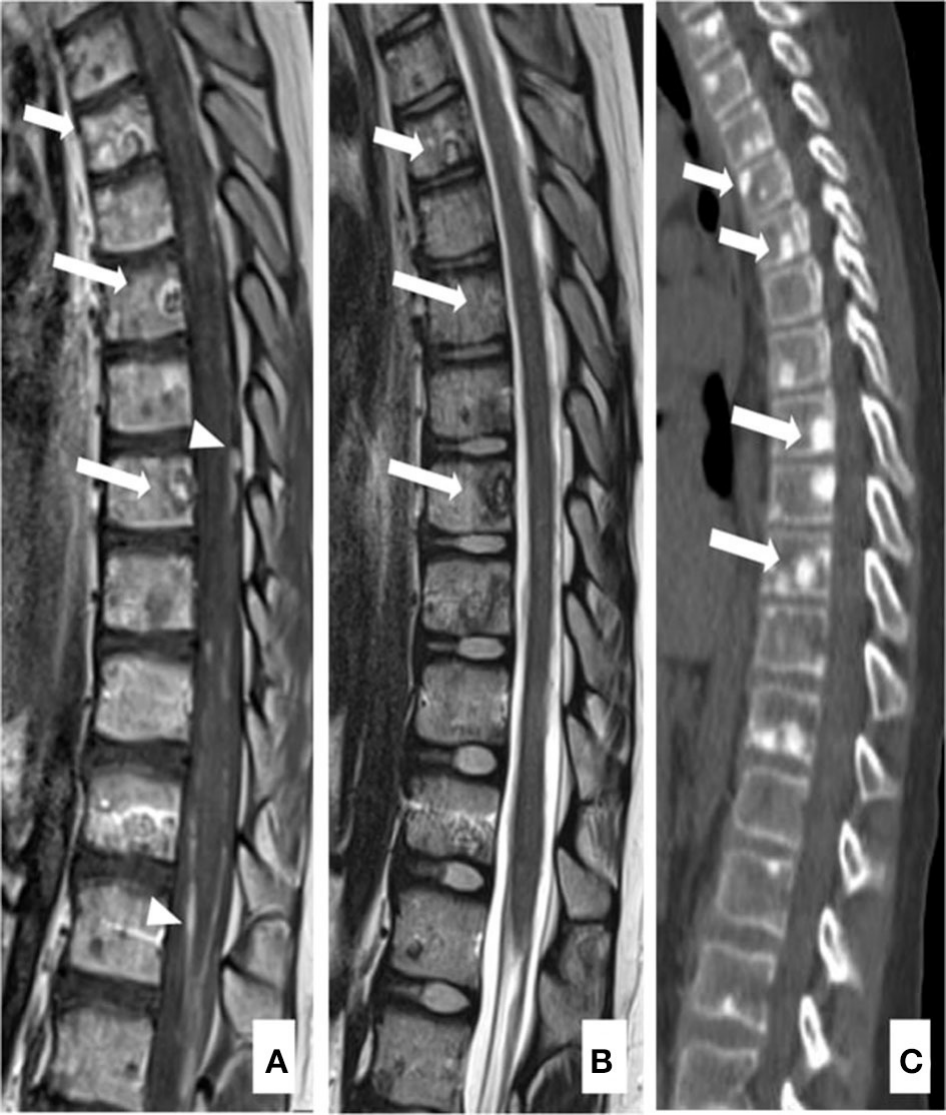
Computed tomography (CT) and magnetic resonance imaging (MRI) of bone metastases of case 1: MRI and CT exams, at 5 months after the diagnosis of DIPG. T1W after gadolinium administration **(A)** and the T2W **(B)** MRI sagittal images show diffuse leptomeningeal peri medullary contrast enhancement nodules [**(A)**, arrowheads] and multiple and diffuse nodules of all the vertebrae with irregular and partial post-contrast enhancement on T1 images [**(A, B)** arrows]. The reformatted sagittal CT images confirm these hyperdense, osteoblastic, rounded lesions involving the vertebrae [**(C)**, arrows].

**Figure 3:**
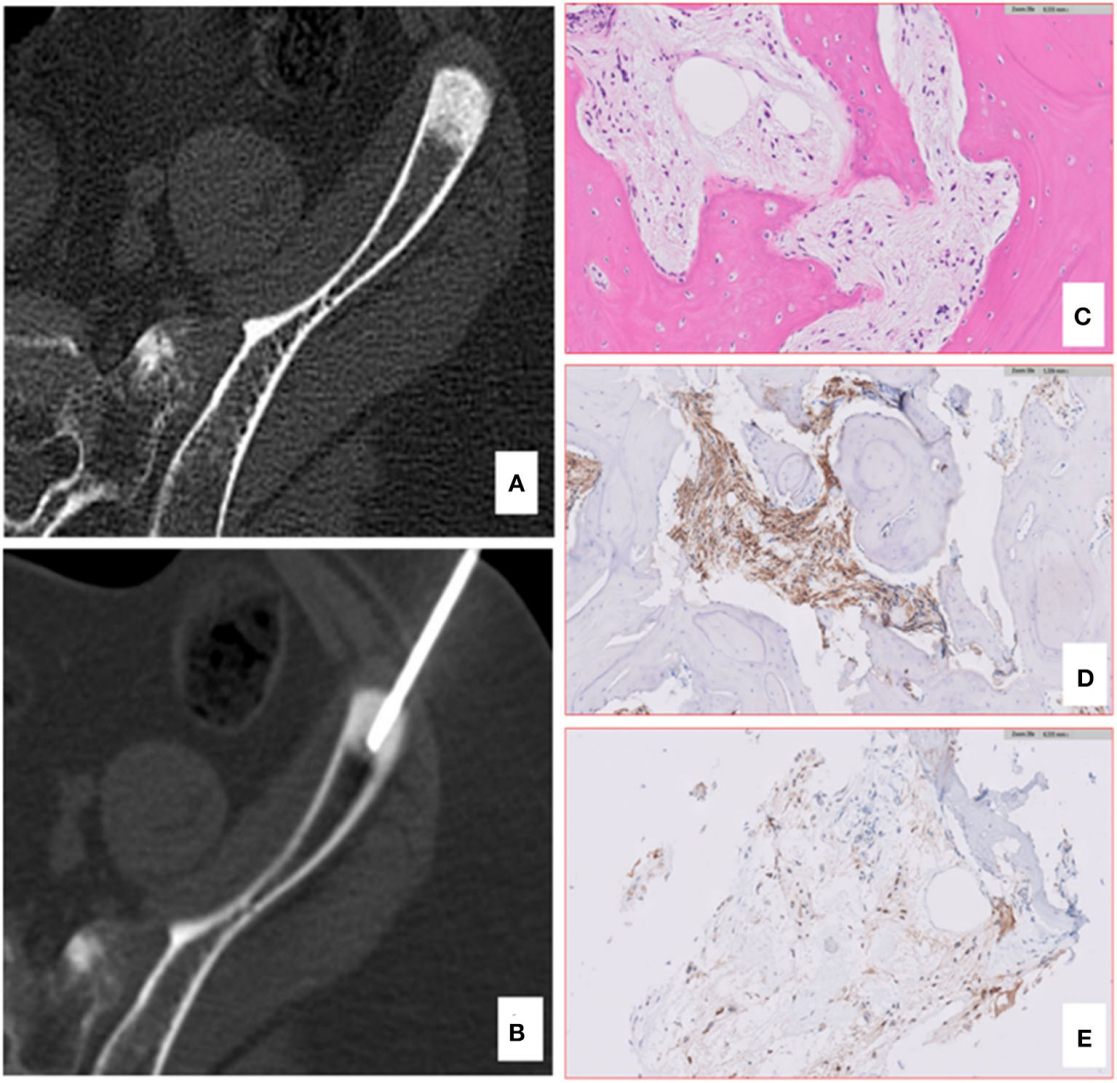
Computed tomography (CT) and pathology of iliac bone lesion of case 1. A bone biopsy was performed using an 11 gauge coaxial bone needle **(A, B)**. The lesion was so stiff that the first needle bent, and a second approach was needed. The bone histology confirmed the skeletal metastases of DIPG **(C)**, showing the presence of a bland spindle cell proliferation infiltrating the lamellar bone with positive IHC staining for GFAP **(D)** and H3K27M-altered protein **(E)**.

An 11-year-old boy was presented in June 2020 with a 1-week history of diplopia due to VI cranial nerve (CN) deficit, headache, and asthenia. Brain computed tomography (CT) scan revealed an enlarged pons characterized by a diffuse hypodense alteration (Figure 1A). Subsequently, MRI of the brain and the spine was performed. The MRI demonstrated the presence of an infiltrative mass involving more than 50% of the pons. MRI features were characteristic and consistent with the diagnosis of DIPG (Figures 1B–F). The MRI excluded other brain and spine lesions referred to metastasis. The patient underwent a stereotactic biopsy of the lesion in accordance with the institutional protocol without complications. Histological examination confirmed the radiological diagnosis of DIPG, showing an infiltrative glial cell proliferation, with tumor cells displaying loss of H3K27me3 and expression of H3K27M-altered protein (Figures 1G–I). In line with the immunohistochemical results, molecular analysis (polymerase chain reaction [PCR] and direct sequencing) documented the presence of H3F3A mutation (c.83A>T, p.K28M); neither activin receptor 1 (ACVR1) nor B-raf proto-oncogene (BRAF) mutations were identified. The patient started a 12-week induction regimen with vinorelbine and nimotuzumab, followed by local radiation therapy (volumetric modulated arc therapy [VMAT]) with 54 Gy in 1.8 Gy per fraction from the third week (Massimino et al., 2014; Massimino, 2022). Five months after the diagnosis of DIPG, the patient presented low back pain, bilateral lower extremity weakness and headache, suggestive of clinical progression. Brain and whole spine MRI demonstrated extensive leptomeningeal enhancement throughout the brain and spinal cord. Enhancing lesions throughout the vertebrae were also noted (Figures 2A–C). Because of the extent of the disease, whole-body CT was performed and revealed numerous osteoblastic lesions involving the vertebrae, sternum, and pelvis. A left iliac bone CT-guided biopsy was performed revealing bone metastases of DIPG (Figures 3A–C). The immunohistochemical evaluation of the malignant cells revealed an expression for GFAP and H3K27M in association with H3K27me3 loss (Figures 3D, E). The clinical course of the disease was rapidly progressive and fatal. The patient died 1 month after the evidence of the metastatic progression.

### 2.2. Case 2

**Figure 4:**
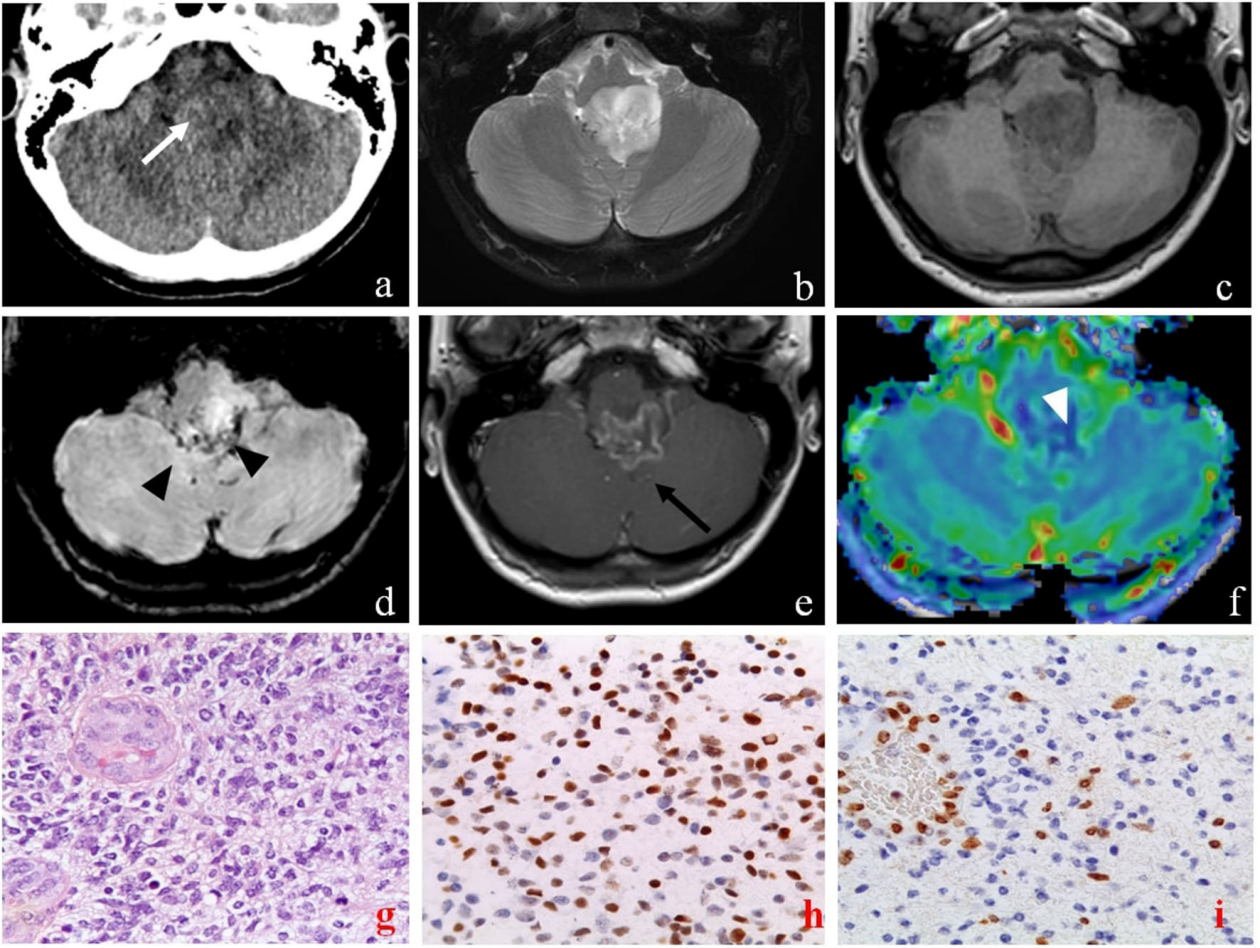
Brain computed tomography (CT), magnetic resonance imaging (MRI), and pathology of primary sites of case 2. The axial CT images show a diffuse low-density lesion centered in the fourth ventricle [**(a)** white arrow]. Axial MRI images confirm the lesion characterized by an inhomogeneous high signal on T2-weighted **(b)** and low signal on T1-weighted **(c)** sequences, extending into the left lateral recess of the fourth ventricle, and compressing the medulla and the cerebellar tonsils. The lesion presents some areas defined by low signal on susceptibility weighted imaging sequences (SWI) due to hemosiderin deposition [**(d)**, black arrowheads], and peripheral enhancement on T1 post-contrast sequences [**(e)**, black arrow]. The MRI also showed peripheral areas of the lesion characterized by high CBV values on perfusion sequences [**(f)**, white arrowhead]. Pathology of primary sites (X40) showed: infiltrative, cellular neoplasm with mitoses and microvascular proliferation [H&E **(g)**]; intense nuclear staining for H3 K27- mutant protein **(h)**; loss of H3 K27me3 expression in tumor cells with retention in endothelial cells **(i)**.

**Figure 5:**
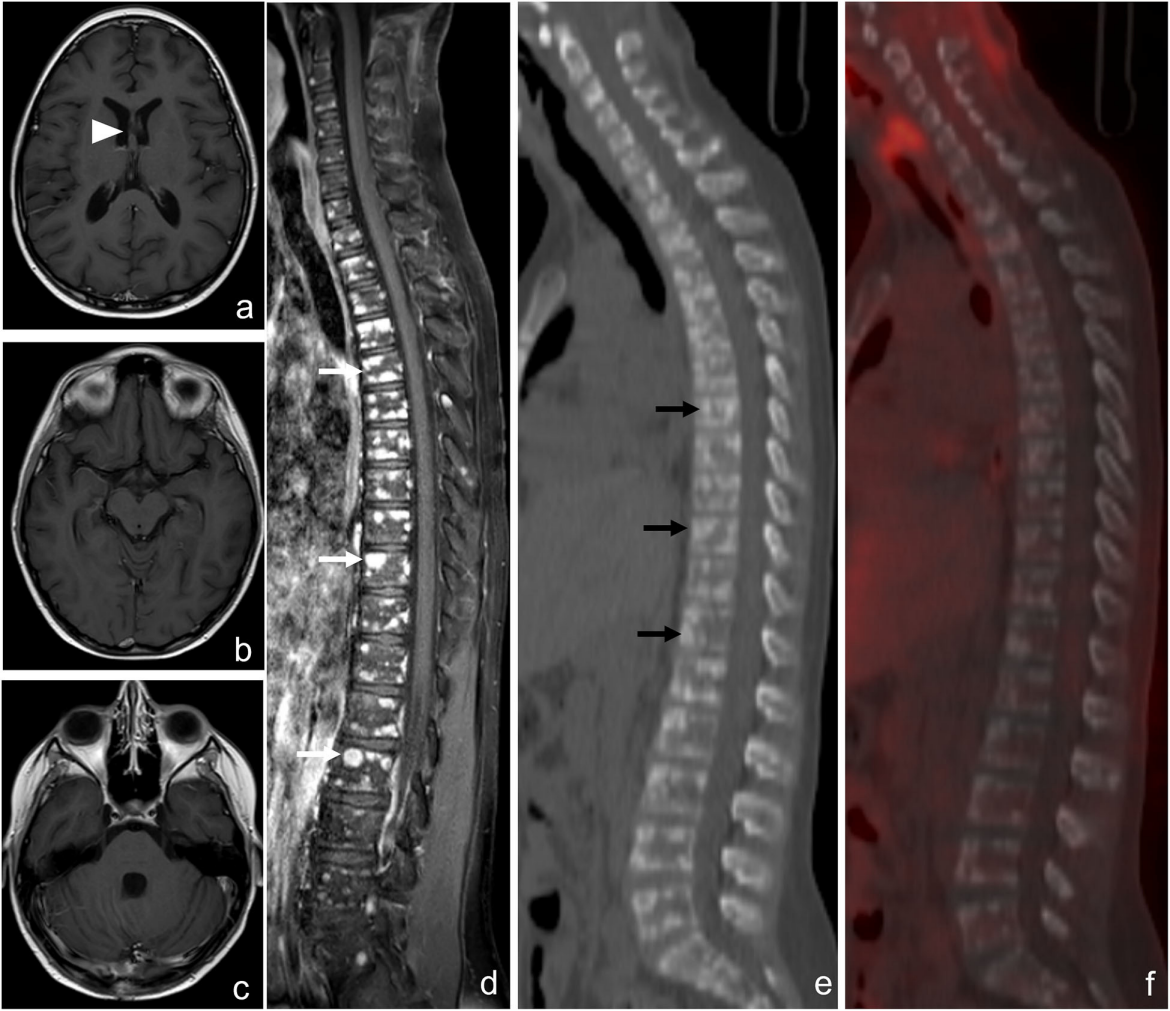
Brain and spine magnetic resonance imaging (MRI) scan and ^18^F-FDG PET-CT after the completion of the second radiotherapy of case 2. Axial contrast-enhanced T1-weighted MRI images show the partial response of the lesion of the septum pellucidum [**(a)**, arrowhead] and the absence of leptomeningeal enhancement **(b, c)**. Spine MRI shows multiple and diffuse nodules of all the vertebrae with irregular contrast enhancement on the T1 image [**(d)**, white arrows]. The reformatted sagittal CT **(e)** and ^18^F-FDG PET-CT **(f)** images confirm hyperdense rounded lesions involving the vertebrae [**(e)**, black arrows] with mild diffuse hypercaptation.

**Figure 6:**
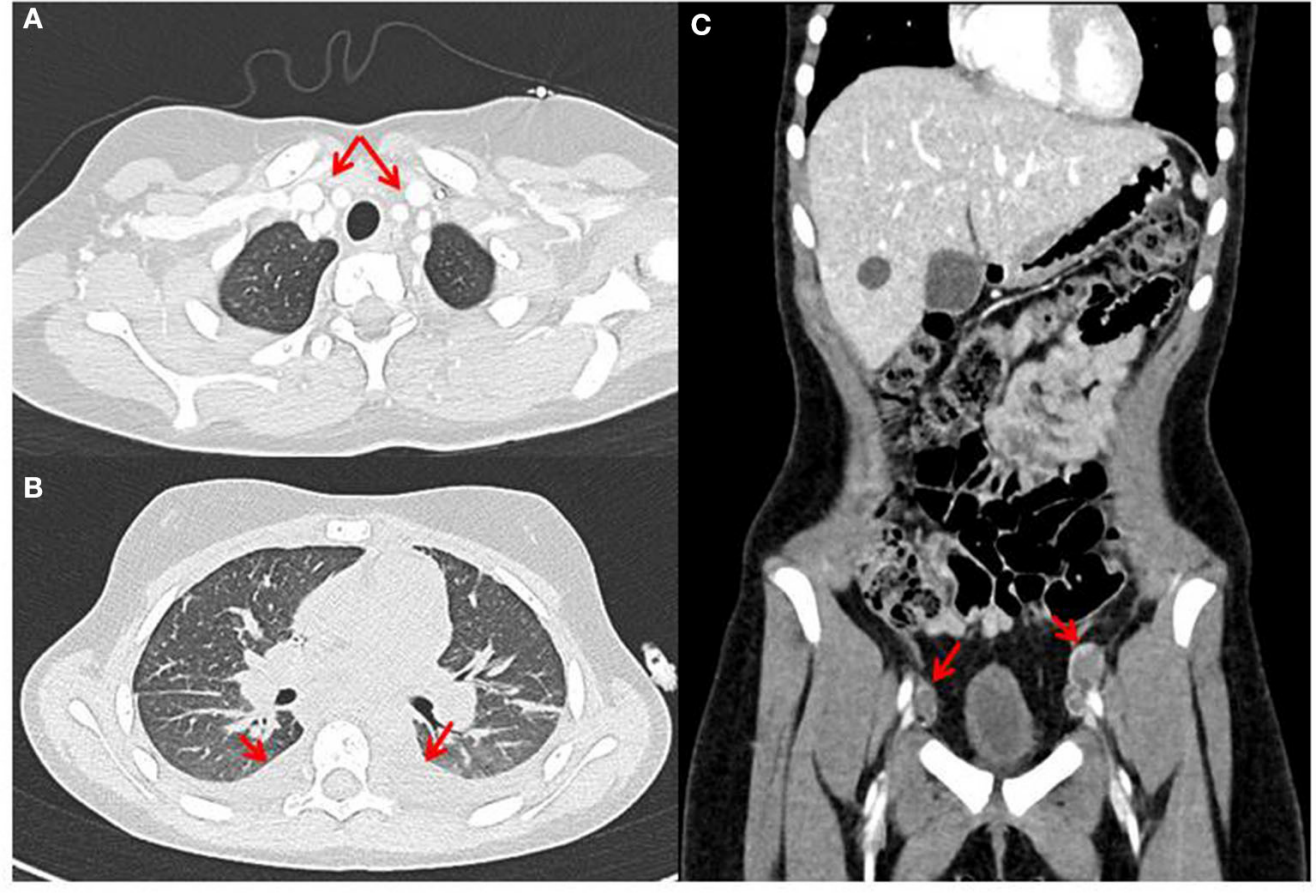
Radiographic features of diffuse midline glioma (DMG), H3K27M-altered (case 2), with multifocal chest **(A)** and abdominal **(B)** lymphadenopathy and pleural effusion **(C)**.

An 11-year-old girl with no relevant family history was presented to our emergency room in March 2020 with a 1-month history of vertiginous syndrome and sporadic vomiting. On examination, horizontal nystagmus and ataxia were documented. An urgent non-enhanced brain CT revealed a large and heterogeneous hypodense mass located within the fourth ventricle, which was slightly dilated (Figure 4a). She was therefore admitted to our hospital. Subsequently, a contrast-enhanced MRI of the brain and whole spine was performed. The MRI confirmed the presence of an infiltrative mass located in the fourth ventricle extending into the left lateral recess (Figures 4b–f). There were no other brain and spine lesions referred to metastases. After multidisciplinary discussion, neuronavigation and occipital craniotomy with tumor resection with direct cortical and subcortical stimulation were performed under general anesthesia. Compared to the first MRI study, MRI scanning within 24 h after surgery documented total resection. Microscopy on tissue sections showed a heterogeneous malignant neoplasm with palisading necrosis and extensive perivascular proliferation. Tumor cells ranged in size from small to medium size, with irregular hyperchromic nuclei and eosinophilic, scarce, or clear cytoplasm arranged in sheets and at the perivascular site (Figure 4g). On immunohistochemical examination, neoplastic cells were positive for vimentin, integrase interactor 1 (INI-1), glial fibrillary acidic protein (GFAP), microtubule-associated protein 2 (MAP2), histone chaperone protein ATRX, and epithelial membrane antigen (EMA). The protein P53 was not expressed. The Ki-67 proliferative index was approximately 40%. The protein H3.3K27me3 was absent, and the expression of H3K27M-altered protein was found (Figures 4h, i). Thus, the pathology was consistent with a diagnosis of pediatric DMG. Whole-exome sequencing (WES) did not reveal targetable mutations. One month after admission, our patient started a 12-week induction regimen with vinorelbine and nimotuzumab, followed by local radiation therapy (VMAT) with 54 Gy in 1.8 Gy per fraction from the third week (Massimino et al., 2014). Three months later, cerebral recurrence involving septum pellucidum, ependyma of lateral ventricles, and leptomeninges was revealed; therefore, she underwent craniospinal radiotherapy (36 Gy in 1.8 Gy per fraction) which was followed by second-line treatment with irinotecan and bevacizumab (IB) for 15 months. A brain and spine MRI scan after the completion of her second radiotherapy showed a partial response of the lesion of the septum pellucidum and of the nodules in the ependyma, absence of leptomeningeal enhancement, and appearance of vertebral lesions (Figures 5a-d). A total body CT revealed diffuse sclerotic vertebral osseous lesions suspected of metastases involving the vertebrae (Figure 5e), ribs, sternum, pelvis, proximal humeri, and proximal femurs. A positron emission tomography with 2-deoxy-2-[fluorine-18]fluoro-D-glucose integrated with computed tomography (^18^F-FDG PET/TC) showed mild diffuse bone hypercaptation; however, multiple biopsies of the lesions were non-diagnostic (Figure 5e). The patient presented good clinical condition, except for mild chronic low back pain responsive to medical treatment. Subsequent MRIs showed brain response but progression of the bone lesions. In October 2021, a brain MRI documented left hemispheric cerebellar recurrence associated with hydrocephalus, and the patient underwent subtotal tumor resection. Next-generation sequencing (NGS) and WES of the tumor confirmed the presence of H3F3A mutation (c.83A>T, p.K28M) and the absence of targetable mutations. At the NGS, additional mutations were found: NBN LOH; NBN deletion; ATR c.5739-14_5739-6delinsT; FGFR4 p.(G388R) c.1162G>A; PTPN11 p.(A72V) c.215C>T. Subsequently, she started third-line treatment with etoposide and temozolomide; however, the clinical course of the disease was slowly progressive. In January 2021, she further developed multifocal chest and abdominal lymphadenopathy and pleural effusion (Figures 6A–C). The pleural fluid analysis did not reveal any cancer cells. However, the droplet digital PCR (ddPCR) performed on pleural effusion identified H3.3A mutation (c.83A>T; p.K28M) confirming the diagnosis of extra-neural metastases. Given the ongoing clinical deterioration, palliative treatment was initiated, and the patient eventually died 24 months after the diagnosis and 3 months after the evidence of metastatic pleural effusion.

## 3. Literature review

A total of 12 cases of extra-neural metastases in DMG have been reported in the literature (Table 1). We excluded from our search cases reported without biopsy or those affected by high grade glioma (HGG), not H3.3 K27-altered. Patients' ages were from 4 to 36 years with a median of 15.5 years (range 4–36) and were predominantly female patients (*n* = 9, 75%). Primary DMGs were mostly in the brain (*n* = 8, 66.7%) and, more rarely, in the spinal cord (*n* = 4, 33.3%) (Megan et al., 2018; Stephens et al., 2019; Bhatt et al., 2020; Handis et al., 2021; Li et al., 2021; Mohiuddin et al., 2021; Al Sharie et al., 2022; Lazow et al., 2022; Silva et al., 2022). The extent of surgical resection was subtotal in four patients (33.3%) (Megan et al., 2018; Stephens et al., 2019; Mohiuddin et al., 2021). Cerebrospinal fluid (CSF) diversion was performed in four patients (33.3%) because of hydrocephalus (Stephens et al., 2019; Mohiuddin et al., 2021; Al Sharie et al., 2022; Silva et al., 2022). Details about cancer treatment were not available in two cases (Megan et al., 2018). Almost all patients underwent adjuvant radiotherapy (*n* = 9, 75%) and chemotherapy (*n* = 8, 66.7%) (Stephens et al., 2019; Handis et al., 2021; Li et al., 2021; Mohiuddin et al., 2021; Lazow et al., 2022; Silva et al., 2022). Because of rapidly declining clinical status, one patient did not initiate any treatment and died of cardiorespiratory failure 2 weeks after presentation (Bhatt et al., 2020). Histological examination was performed in 11 patients (91.7%) (Megan et al., 2018; Stephens et al., 2019; Bhatt et al., 2020; Handis et al., 2021; Li et al., 2021; Mohiuddin et al., 2021; Al Sharie et al., 2022; Lazow et al., 2022; Silva et al., 2022; Aftahy et al., 2023). In one patient, the diagnosis was made on CSF by NGS, which revealed a HIST1H3B mutation (Li et al., 2021). At diagnosis, brain and/or spinal metastases were present in eight patients (66.7%), while tumor spread outside the CNS was present in seven patients (58.3%) (Megan et al., 2018; Stephens et al., 2019; Bhatt et al., 2020; Handis et al., 2021; Li et al., 2021; Mohiuddin et al., 2021; Al Sharie et al., 2022; Lazow et al., 2022; Silva et al., 2022; Aftahy et al., 2023). Extra-neural metastases were detected after the diagnosis in the remaining five cases (41.7%) (Megan et al., 2018; Stephens et al., 2019; Bhatt et al., 2020; Handis et al., 2021; Li et al., 2021; Mohiuddin et al., 2021; Al Sharie et al., 2022; Lazow et al., 2022; Silva et al., 2022; Aftahy et al., 2023). In eight patients (66.7%), DMG metastasized in a single extra-neural site (seven bones, one peritoneum) (Megan et al., 2018; Stephens et al., 2019; Bhatt et al., 2020; Handis et al., 2021; Li et al., 2021; Mohiuddin et al., 2021; Al Sharie et al., 2022; Lazow et al., 2022; Silva et al., 2022; Aftahy et al., 2023). In the remaining cases (*n* = 4, 33.3%), multiple extra-neural locations were detected involving bones, lymph nodes, lungs, soft tissue, peritoneum, lungs, and pleura (Mohiuddin et al., 2021; Lazow et al., 2022; Silva et al., 2022). Extra-neural metastases were treated with palliative locoregional radiotherapy in only one patient (8.3%) and with systemic chemotherapy in two patients (16.7%) (Mohiuddin et al., 2021; Silva et al., 2022; Aftahy et al., 2023). At the last follow-up, all patients died with a median overall survival (OS) from primary DMG diagnosis of 9 months (range, 0.5–15), and a median OS from extra-neural metastases occurrence of 6 months (range, 0.5–16) (Megan et al., 2018; Stephens et al., 2019; Bhatt et al., 2020; Handis et al., 2021; Li et al., 2021; Mohiuddin et al., 2021; Al Sharie et al., 2022; Lazow et al., 2022; Silva et al., 2022; Aftahy et al., 2023).

**Table 1:** Diffuse midline glioma, H3 K27a studies reporting extra-neural metastases.

**Pt**	**Age (years)/sex**	**Diagnosis**	**Site of tumor**	**CNS metastases**	**Timing of appearance since diagnosis**	**Extra-CNS metastases**	**Timing of appearance since diagnosis**	**Surgery**	**Radiotherapy**	**Chemotherapy**	**OS (ms)**	**References**
1	11/M	DMG H3F3A K27mt (WES)	Left lateral ventricle, hypothalamus, fornices, and left midbrain	None	–	Bones	3.5 months	Subtotal resection	NA	NA	9	Megan et al., 2018
2	12/F	DMG H3F3A K27mt (WES)	Tectal/pineal gland	Spinal cord	At diagnosis	Bones	5.5 months	Subtotal resection	NA	NA	13	Megan et al., 2018
3	15/F	DMG H3K27mt (IHC)	Spine (T12-L1, conus medullaris)	Multifocal brain	At diagnosis	Bones, bone marrow	At diagnosis	Biopsy of the thoracic spinal mass	None	None	0.5	Bhatt et al., 2020
4	4/F	DMG H3F3A K27mt (IHC, NGS)	Brainstem	Spine	1 month	Peritoneum	14 months since diagnosis	Subtotal resection + VPS	First line Focal RT 54 Gy + CSI (+ boost to S1 metastasis) 50.4 Gy At progression Skull base-mid lumbar spine 50.4 Gy Cranial disease 35 Gy S1 disease 15 Gy	First line Temozolomide	15	Stephens et al., 2019
5	36/F	DMG HIST1H3B K27mt (NGS)	Pons	CSF	At diagnosis	Bones	At diagnosis	None (liquid biopsy for diagnosis)	First line Focal RT 54 Gy	First line Temozolomide	13	Li et al., 2021
6	20/F	DMG H3F3A K27M (NGS)	Right thalamus	Intracranial and spinal leptomeninges	4–6 months	Bones	4–6 months	Subtotal resection	First line Focal RT 60 Gy At progression Proton RT Midbrain-spinal axis 36 Gy + Boost to spinal disease cumulative dose of 45 Gy	First line Temozolomide At progression Bevacizumab (10 mg/kg) every 2 weeks 2. Panobinostat 30 mg/3 days a week every other week in combination with bevacizumab	11	Mohiuddin et al., 2021
7	17/F	DMG H3F3A K27M (NGS)	Left hippocampus extending into the left posterior midbrain	Intracranial and spine leptomeninges	At diagnosis	Chest, abdomen, and pelvis lymph nodes, lung, pleura, liver, and omental fat stranding	4 months	Biopsy of the brain lesion + VPS	First line CSI 39.6 Gy + Focal boost 18 Gy	First line Temozolomide	5	Mohiuddin et al., 2021
8	16/F	DMG H3F3A K27M (NGS)	Spine (T7-L3)	Intracranial pial and parenchymal disseminations	At diagnosis	Bones	At diagnosis	Open biopsy of spinal lesion	First line Whole cranial and vertebral axis	First line Vincristine (1.5 mg/m^2^ once daily), procarbazine (100 mg/m2 once daily), lomustine (100 mg/m^2^ once daily), cyclophosphamide (1000 mg/m^2^ once daily)	5	Handis et al., 2021
9	8/F	DMG H3K27-altered with EZHIP overexpression (NGS)	Pons, thalamus, and bilateral temporal lobes	None	–	Muscle, peritoneum, infratemporal fossa, and along the lumbosacral nerve roots	At diagnosis (muscle, brachial plexus, lumbosacral nerve roots) 4 months (peritoneum)	Biopsy of the lateral rectus muscle + biopsy of the temporal lobe + VPS	First line Proton RT CSI (+right orbit) 52.2 Gy + Brachial plexus 46.5 Gy	None	NA	Silva et al., 2022
10	12/F	DMG H3F3A K27M (NGS)	Periventricular white matter, temporal structures, optic chiasm, brainstem, septum pellucidum, cerebellum	Spine	At diagnosis	Bones, lungs	At diagnosis (bones) Later (lungs)	Biopsy of thoracic spinal lesion + Bone biopsy	First line CSI 45 Gy	At progression Cabozantinib 40 mg/m^2^ daily for 28-day cycles	9	Lazow et al., 2022
11	19/M	DMG H3K27 altered	Spine (D11-L1)	Intracranial and spine leptomeninges	At diagnosis	Bones	At diagnosis	Biopsy of bone lesion + VPS	First line D11-L1 5.4 Gy CSI 39.6 Gy Boost to the suprasellar mass 14.4 Gy	First line Temozolomide	7	Al Sharie et al., 2022
12	24/M	DMG H3K27mt (IHC)	Spine (C5-D7)	Brain and spine	At diagnosis	Bones (vertebrae, sternum)	At diagnosis	Biopsy of brain lesion + bone biopsy	First line CSI (included vertebrae) 36 Gy	First line Temozolomide	3	Aftahy et al., 2023
13	11/M	DMG H3K27M (IHC, NGS)	Pons	Brain and spinal leptomeninges	5 months	Bones	5 months	Biopsy of pons	First line Focal RT 54 Gy	First line Nimotuzumab (150 mg/m^2^) and Vinorelbine (20 mg/m^2^) every week	6	Present case
14	11/F	DMG H3K27M (IHC, NGS)	Cerebellum	Septum pellucidum, lateral ventricles, leptomeninges	6 months	Bones, chest, abdomen, and pelvis lymph nodes pleura	6 months (bone) 23 months (lymph nodes and pleura)	Total resection of the cerebellar mass	First line Focal RT 54 Gy At progression CSI 36 Gy CFP 28 Gy	First line Nimotuzumab (150 mg/m^2^) and Vinorelbine (20 mg/m^2^) every week At progression Bevacizumab (10 mg/kg) + Irinotecan (125 mg/m^2^) every 2 weeks 2. Etoposide + Temozolomide	24	Present case

M, male; F, female; DMG, diffuse midline glioma; mt, mutant; a, altered; IHC, immunohistochemistry; NGS, next-generation sequencing; RT, radiotherapy; CSI, craniospinal irradiation; Gy, gray.

## 4. Discussion

Metastases of pediatric DMG outside the CNS are extremely rare. Since “diffuse midline glioma, H3K27M- mutant” was introduced as a distinct entity in the 2016 edition of the WHO classification of tumors of CNS, a total of 12 cases (3 M) aged 4–36 years of extra-neural metastases have been reported in the literature (Table 1). We presented an 11-year-old boy with DIPG and an 11-year-old girl with cerebellum DMG who developed extra-neural metastases a few months after diagnosis, despite cancer treatment consisting of radiotherapy and concomitant nimotuzumab and vinorelbine (Massimino et al., 2014; Massimino, 2022). Localization of DMG in midline sites such pineal region, hypothalamus, and cerebellum is exceptional (Solomon et al., 2016; Meyronet et al., 2017; Nakata et al., 2017). In a series of 47 diffuse midline gliomas with histone H3K27M mutation, only one 9-year-old boy presented with a tumor arising in the cerebellum (Solomon et al., 2016). Similarly, in a retrospective series of 164 cases with molecularly confirmed H3K27M-mt DMGs, the cerebellum location was identified in only two patients (Zheng et al., 2022). Interestingly, Wang et al. (2018) showed that patients with H3K27M-mutant gliomas in unusual anatomical locations (cerebellum, corpus callosum, lateral ventricle, frontal, and temporal lobe) had a better prognosis compared with those with corresponding tumors in the brainstem. Recently, Hazaymeh et al. (2022) showed that patients affected by glioblastoma multiforme (GBM) undergoing gross total resection exhibited a significant survival benefit compared to their counterparts without gross total resection. Our cases 1 and 2 developed osseous metastases 5 months and 6 months, respectively, since the initial diagnosis. Osseous and bone marrow metastases of our case 1 were documented by CT-guided biopsy of the left iliac bone, and the patient died 1 month later. It is interesting to note that bone metastases are common sites of extra-neural involvement in high-grade gliomas (HGGs), such as DMG and GBM. This predilection of bone may come from both tumor-derived and extracellular niche-derived cues. For example, many hematopoietic stem cell proteins are expressed by GBM cells, including stromal cell-derived factor 1 alpha (SDF-1α), C-X-C chemokine receptor type 4 (CXCR4), osteopontin (OPN), and cathepsin K (CATK) (Hira et al., 2018). Glioblastoma cells are also able to recruit bone marrow-derived progenitor cells providing a perivascular support role regulated by vascular endothelial growth factor (VEGF) (Burrell et al., 2014). In addition, CXCR4, OPN, CATK, and CD44 are induced by hypoxia-inducible factor-1α and VEGF, two proteins known to increase glioma aggressiveness and invasion (Colwell et al., 2017). In addition to bone metastases, our case 2 developed lymph nodes and pleura involvement. The diagnosis of multiple extra-neural metastases was more difficult since bone and lymph node biopsies and pleural fluid analysis were negative for cancer cells. The diagnosis was concluded due to positive ddPCR for H3.3A mutation on pleural effusion. Recently, Wolter et al. (2022) reported on the application and validation of a set of molecular assays for glioma diagnostics based on ddPCR, enabling the detection of diagnostically relevant glioma-associated mutations in the isocitrate dehydrogenase (IDH)1, IDH2, H3-3A, BRAF, and protein kinase C alpha (PRKCA) genes, as well as in the telomerase (TERT) promoter and other relevant copy number alterations. Recently, Massimino et al. identified and validated a prognostic marker based on the expression of 13 circulating microRNAs in serum that can shed light on a patient's risk of progression (Iannó et al., 2022).

Due to its rarity, the biological mechanisms behind tumor dissemination outside the CNS of DMG have not been well-described. Although improved care of patients affected by DMG is going to lead in some cases to longer survival, extra-neural metastases in DMG were detected at diagnosis or relatively early after diagnosis. Certainly, the absence of routine surveillance for extra-neural metastases in the staging of DMG may contribute to the underreporting of metastatic disease. Although recent studies have suggested that CSF shunting could be a risk factor for disseminated disease, a recent comparative outcome multivariate analysis showed that the ventricular route is not a likely pathway for the spread of leptomeningeal disease or for distant tumor recurrences (Mistry et al., 2019). Increasing evidence from clinical and experimental studies suggests that surgical trauma caused by biopsies or resections may potentially lead to tumor progression and metastatic disease (Alieva et al., 2018). On hospital admission, both our patients underwent neurosurgical procedures: case 1 a stereotactic biopsy of the pontine lesion and case 2 a complete surgical resection of cerebellum mass. Recently, Massimino et al. showed that pediatric patients affected with pediatric DMG undergoing biopsies had more dissemination (*P* = 0.04) and less local progression (Massimino et al., 2014). Alieva et al. (2018) showed, in a retrospective analysis of GBM patients, an increase in tumor volume after biopsy. In mice, the cellular mechanisms mediating this response are dependent on inflammation, especially on the CCL-2-dependent recruitment of macrophages, which can be blocked by treatment with dexamethasone. The immune system may also have a role in tumor extra-neural dissemination. In patients affected by GBM, multiple hypotheses have been postulated as why, such aggressive tumors, only rarely exhibit metastases outside the brain. The main pathophysiological ideas are the “seed vs. soil” hypothesis (Mohme et al., 2017) that describes the preference of metastatic tumor cells to grow inside the brain where the local microenvironment is favorable, and the “peripheral immunosurveillance” hypothesis, which holds that the activated peripheral immune system is able to eliminate GBM tumor cells that left the immune protected brain microenvironment. However, the discovery of circulating tumor cells (CTCs) in up to 20% of GBM patients has renewed interest in this discussion. However, it remains unclear why the CTCs do not form extra-neural metastases at the expected frequency. The occurrence of extracranial GBM metastases in recipients of organ transplantation from donors diagnosed with GBM points to a decisive role of the immune system in containing extracranial growth (Jimsheleishvili et al., 2014; Nauen and Li, 2014). Recently, Mohme et al. (2020) described a case of extracranial metastases from GBM during immunological remission of the intracerebral tumor with checkpoint inhibition. They postulated that the combination of functional impairment of the peripheral immune system, as reflected by a steady increase of exhaustion markers and the occurrence of metastasis with an increased mutational burden, enabled the extracranial dissemination and disease progression, while intracranial GBM could be controlled by checkpoint inhibition (Mohme et al., 2020). Similarly, our case 2 showed disease progression at bone sites and partial response of intracranial DMG to second-line treatment with craniospinal irradiation and systemic chemotherapy with irinotecan and bevacizumab regimen. At the time, neither an international nor European consensus chemotherapy regimen was universally agreed upon for DMG treatment, and the Phase 2 Children's Oncology Group (COG) study ACNS0126 demonstrated that single-agent TMZ during and after radiotherapy failed to improve pediatric HGG survival compared to historical controls (Jakacki et al., 2016). In Italy, the standard treatment of pediatric H3K27M-a DMG includes radiotherapy, concomitant nimotuzumab and vinorelbine, and re-irradiation at relapse (Massimino, 2022). Recent studies support the bevacizumab regimen showing superior survival compared to historical-agent regimens and nearly all other published treatment strategies (Hummel et al., 2016; Lu et al., 2019). However, further research is needed to prove the safety and efficacy of bevacizumab in children and adolescents affected by DMG, H3 K27-altered.

## 5. Limitations

Our study have some limitations, primarily due to the paucity of extra-neural DMG metastases in the literature. All the studies included in this review are case reports or case series. None of our patients completed genetic testing for germline mutations; therefore, familial predisposition cancer syndrome cannot be excluded.

## 6. Conclusion

Extra-neural metastasis of DMG is a rare event. The present cases emphasize the need to consider unusual localization of pediatric brain cancers, especially because early diagnosis and active treatment may be crucial to improve prognosis and survival. Even if no standard therapy exists to treat extra-neural metastasis of DMG, it is mandatory to establish promptly an accurate and specific diagnosis in order to develop a personalized plan of treatment. Moreover, the biopsy of the primary and metastatic sites should be considered for any pediatric patients with DMGs due to the important prognostic implications as well as to develop more effective treatment strategies. Considering the rarity of extra-neural metastasis of DMGs, international registries and collaborative multicenter studies are warranted to gain further insights into the molecular pathology of DMG, H3K27-altered and improve the quality of life and the final outcome of patients with this deadly disease.

## Data availability statement

The original contributions presented in the study are included in the article/supplementary material, further inquiries can be directed to the corresponding author.

## Ethics statement

Written informed consent was obtained from the individual(s), and minor(s)' legal guardian/next of kin, for the publication of any potentially identifiable images or data included in this article.

## Author contributions

LD and LQ: conceptualization and methodology. LD, MP, AC, GS, GSC, and PS: data curation. MM and AM: formal analysis. LQ, LD, SP, GS, GC, ME, MM, AM, and NN: investigation. GC: resources. MM and LQ: supervision. LD, ME, GSC, AC, and CR: writing—original draft. SP, AM, NN, GC, and MM: writing—review and editing. All authors have read and agreed to the published version of the manuscript.
